# 2-Acetonyl-2-hydroxy­indan-1,3-dione

**DOI:** 10.1107/S1600536809016067

**Published:** 2009-05-07

**Authors:** Hoong-Kun Fun, Ching Kheng Quah, Mehtab Parveen, Raza Murad Ghalib, Sayed Hasan Mehdi

**Affiliations:** a X-ray Crystallography Unit, School of Physics, Universiti Sains Malaysia, 11800 USM, Penang, Malaysia; b Department of Chemistry, Aligarh Muslim University, Aligarh 202 002, Uttar Pradesh, India

## Abstract

In the title compound, C_12_H_10_O_4_, the five-membered ring adopts an envelope conformation, with the C*sp* ^3^ atom at the flap [deviation = 0.145 (2) Å]. In the crystal structure, mol­ecules are linked by inter­molecular O—H⋯O and C—H⋯O hydrogen bonds, forming a three-dimensional network.

## Related literature

For the activities and applications of ninhydrin derivatives, see: Ruhemann (1910 ▶); Kaiser *et al.* (1970 ▶). For bond-length data, see: Allen *et al.* (1987 ▶). For the stability of the temperature controller used for the data collection, see: Cosier & Glazer (1986 ▶).
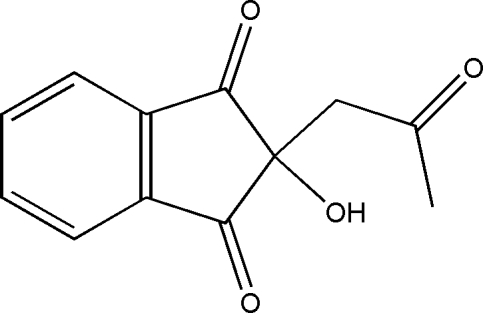


## Experimental

### 

#### Crystal data

C_12_H_10_O_4_
*M* *_r_* = 218.20
Orthorhombic, 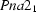

*a* = 18.1190 (2) Å
*b* = 8.8135 (1) Å
*c* = 6.2585 (1) Å
*V* = 999.43 (2) Å^3^
*Z* = 4
Mo *K*α radiation
μ = 0.11 mm^−1^
*T* = 100 K
0.29 × 0.19 × 0.08 mm

#### Data collection

Bruker SMART APEXII CCD area-detector diffractometer
Absorption correction: multi-scan (**SADABS**; Bruker, 2005 ▶) *T*_min_ = 0.969, *T*_max_ = 0.992
14417 measured reflections
1818 independent reflections
1720 reflections with *I* > 2σ(*I*)
*R*_int_ = 0.034

#### Refinement

*R*[*F* ^2^ > 2σ(*F* ^2^)] = 0.033
*wR*(*F* ^2^) = 0.106
*S* = 1.18
1818 reflections
150 parameters
1 restraint
H atoms treated by a mixture of independent and constrained refinement
Δρ_max_ = 0.42 e Å^−3^
Δρ_min_ = −0.24 e Å^−3^

### 

Data collection: *APEX2* (Bruker, 2005 ▶); cell refinement: *SAINT* (Bruker, 2005 ▶); data reduction: *SAINT*; program(s) used to solve structure: *SHELXTL* (Sheldrick, 2008 ▶); program(s) used to refine structure: *SHELXTL*; molecular graphics: *SHELXTL*; software used to prepare material for publication: *SHELXTL* and *PLATON* (Spek, 2009 ▶).

## Supplementary Material

Crystal structure: contains datablocks global, I. DOI: 10.1107/S1600536809016067/ci2791sup1.cif

Structure factors: contains datablocks I. DOI: 10.1107/S1600536809016067/ci2791Isup2.hkl

Additional supplementary materials:  crystallographic information; 3D view; checkCIF report

## Figures and Tables

**Table 1:** Hydrogen-bond geometry (Å, °)

*D*—H⋯*A*	*D*—H	H⋯*A*	*D*⋯*A*	*D*—H⋯*A*
O3—H1*O*3⋯O2^i^	0.86 (3)	1.93 (3)	2.7907 (16)	174 (3)
C3—H3*A*⋯O4^ii^	0.93	2.51	3.401 (2)	159
C12—H12*A*⋯O4^iii^	0.96	2.54	3.408 (2)	150

Symmetry codes: (i) 
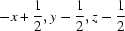
; (ii) 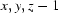; (iii) 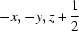.

**Fig. 1.**
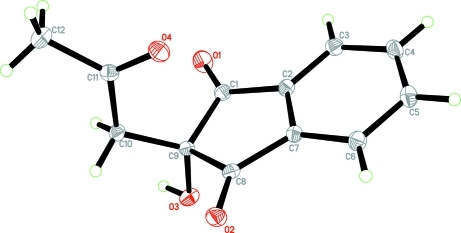
The molecular structure of the title compound, showing 50% probability displacement ellipsoids and the atom-numbering scheme.

**Fig. 2.**
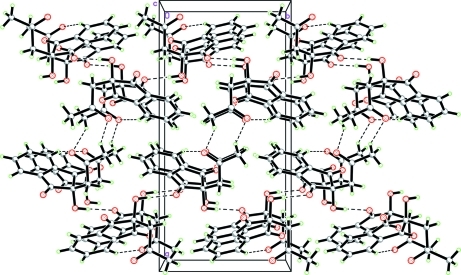
The crystal packing of the title compound, viewed along the c axis. Intermolecular hydrogen bonds are shown as dashed lines.

## supplementary crystallographic information

### Comment

Ninhydrin is used to detect α-amino acids, proteins and dipeptides. When it reacts with free amines, a deep blue or purple colour known as Ruhemann's purple (RP) is evolved (Ruhemann, 1910). Ninhydrin is also used to monitor deprotection in solid phase peptide synthesis (Kaiser Test) (Kaiser *et al.*, 1970). It is one of the most widely used reagents for chemical development of fingerprints on porous surfaces. We herein present the crystal structure of the title compound, a derivative of ninhydrin.

Bond lengths (Allen *et al.*, 1987) and angles in the title molecule (Fig. 1) are within normal ranges. The indan ring system (C1-C9) is almost planar, with a maximum deviation of 0.072 (1) Å for atom C9 while the dihedral angle formed by the benzene ring and the five-membered ring is 1.87 (8)°. The keto atom O1 lies 0.075 (2) Å from the indan plane whereas the keto atom O2 is displaced from the C1-C9 plane by 0.184 (2) Å. The five-membered ring adopts an envelope conformation, with atom C9 at the flap [deviation 0.145 (2) Å]. The C2—C1—C9—O3 torsion angle is 103.16 (14) Å.

In the crystal structure (Fig. 2), the molecules are linked by intermolecular O3—H1O3···O2 and C3—H3A···O4 hydrogen bonds (Table 1) into a two-dimensional network parallel to the (100). The adjacent networks are linked via C12—H12A···O4 hydrogen bonds to form a three-dimensional network.

### Experimental

The title compound was synthesized by the reaction of ninhydrin (1.78 g), trichloroacetic acid (1.64 g) and catalytic amount of magnesium in presence of acetone. Ninhydrin and tricholoro acetic acid in molar ratio 1:1 were allowed to reflux with acetone in presence of Mg turnings for 1 h. The reaction mixture was dried under reduced pressure and was purified by chromatography over silica gel column. Elution of the column with petroleum ether-diethyl ether (4:1) followed by crystallization with petroleum ether-chloroform (1:1) afforded fine crystals of the title compound (120 mg, m.p. 399 K).

### Refinement

Atom H1O3 was located in a difference Fourier map and refined freely. The remaining H atoms were positioned geometrically and refined using a riding model, with C-H = 0.93–0.97 Å and *U*_iso_(H) = 1.2 and 1.5 *U*_eq_(C). A rotating-group model was applied for the methyl group. In the absence of significant anomalous dispersion, 1513 Friedel pairs were merged for the final refinement.

### Crystal data

**Table:**

C_12_H_10_O_4_	*F*(000) = 456
*M**_r_* = 218.20	*D*_x_ = 1.450 Mg m^−^^3^
Orthorhombic, *P**n**a*2_1_	Mo *K*α radiation, λ = 0.71073 Å
Hall symbol: P 2c -2n	Cell parameters from 5199 reflections
*a* = 18.1190 (2) Å	θ = 3.2–31.5°
*b* = 8.8135 (1) Å	µ = 0.11 mm^−^^1^
*c* = 6.2585 (1) Å	*T* = 100 K
*V* = 999.43 (2) Å^3^	Plate, yellow
*Z* = 4	0.29 × 0.19 × 0.08 mm

### Data collection

**Table:**

Bruker SMART APEXII CCD area-detector diffractometer	1818 independent reflections
Radiation source: fine-focus sealed tube	1720 reflections with *I* > 2σ(*I*)
graphite	*R*_int_ = 0.034
φ and ω scans	θ_max_ = 31.7°, θ_min_ = 2.3°
Absorption correction: multi-scan (*SADABS*; Bruker, 2005)	*h* = −26→26
*T*_min_ = 0.969, *T*_max_ = 0.992	*k* = −12→13
14417 measured reflections	*l* = −9→9

### Refinement

**Table:**

Refinement on *F*^2^	Primary atom site location: structure-invariant direct methods
Least-squares matrix: full	Secondary atom site location: difference Fourier map
*R*[*F*^2^ > 2σ(*F*^2^)] = 0.033	Hydrogen site location: inferred from neighbouring sites
*wR*(*F*^2^) = 0.106	H atoms treated by a mixture of independent and constrained refinement
*S* = 1.18	*w* = 1/[σ^2^(*F*_o_^2^) + (0.0696*P*)^2^ + 0.0468*P*] where *P* = (*F*_o_^2^ + 2*F*_c_^2^)/3
1818 reflections	(Δ/σ)_max_ = 0.001
150 parameters	Δρ_max_ = 0.42 e Å^−^^3^
1 restraint	Δρ_min_ = −0.24 e Å^−^^3^

### Special details

**Table:**

Experimental. The crystal was placed in the cold stream of an Oxford Cyrosystems Cobra open-flow nitrogen cryostat (Cosier & Glazer, 1986) operating at 100.0 (1) K.
Geometry. All e.s.d.'s (except the e.s.d. in the dihedral angle between two l.s. planes) are estimated using the full covariance matrix. The cell e.s.d.'s are taken into account individually in the estimation of e.s.d.'s in distances, angles and torsion angles; correlations between e.s.d.'s in cell parameters are only used when they are defined by crystal symmetry. An approximate (isotropic) treatment of cell e.s.d.'s is used for estimating e.s.d.'s involving l.s. planes.
Refinement. Refinement of *F*^2^ against ALL reflections. The weighted *R*-factor *wR* and goodness of fit *S* are based on *F*^2^, conventional *R*-factors *R* are based on *F*, with *F* set to zero for negative *F*^2^. The threshold expression of *F*^2^ > σ(*F*^2^) is used only for calculating *R*-factors(gt) *etc*. and is not relevant to the choice of reflections for refinement. *R*-factors based on *F*^2^ are statistically about twice as large as those based on *F*, and *R*- factors based on ALL data will be even larger.

### Fractional atomic coordinates and isotropic or equivalent isotropic displacement parameters (Å^2^)

**Table:**

	*x*	*y*	*z*	*U*_iso_*/*U*_eq_	
O1	0.13189 (7)	0.07160 (14)	0.5143 (2)	0.0217 (3)	
O2	0.21514 (6)	0.36656 (13)	1.0967 (2)	0.0158 (2)	
O3	0.27231 (6)	0.16183 (13)	0.7536 (2)	0.0153 (2)	
O4	0.06202 (6)	0.15803 (14)	1.0224 (2)	0.0165 (3)	
C1	0.14680 (8)	0.17868 (17)	0.6278 (3)	0.0130 (3)	
C2	0.12208 (8)	0.33840 (16)	0.6024 (3)	0.0121 (3)	
C3	0.07836 (8)	0.40053 (18)	0.4425 (3)	0.0145 (3)	
H3A	0.0609	0.3415	0.3301	0.017*	
C4	0.06148 (8)	0.55486 (19)	0.4566 (3)	0.0161 (3)	
H4A	0.0314	0.5991	0.3535	0.019*	
C5	0.08914 (9)	0.64417 (18)	0.6239 (3)	0.0163 (3)	
H5A	0.0784	0.7473	0.6273	0.020*	
C6	0.13236 (8)	0.58104 (17)	0.7850 (3)	0.0144 (3)	
H6A	0.1502	0.6401	0.8967	0.017*	
C7	0.14801 (8)	0.42619 (16)	0.7731 (3)	0.0115 (3)	
C8	0.18969 (8)	0.32969 (16)	0.9244 (3)	0.0115 (3)	
C9	0.19764 (8)	0.17061 (16)	0.8252 (3)	0.0107 (3)	
C10	0.17882 (8)	0.04091 (17)	0.9758 (3)	0.0125 (3)	
H10A	0.1889	−0.0547	0.9049	0.015*	
H10B	0.2103	0.0470	1.1008	0.015*	
C11	0.09891 (8)	0.04343 (17)	1.0464 (3)	0.0121 (3)	
C12	0.06798 (9)	−0.10028 (18)	1.1373 (3)	0.0173 (3)	
H12A	0.0295	−0.0763	1.2371	0.026*	
H12B	0.1064	−0.1552	1.2092	0.026*	
H12C	0.0482	−0.1614	1.0240	0.026*	
H1O3	0.2777 (12)	0.069 (3)	0.713 (5)	0.029 (7)*	

### Atomic displacement parameters (Å^2^)

**Table:**

	*U*^11^	*U*^22^	*U*^33^	*U*^12^	*U*^13^	*U*^23^
O1	0.0303 (6)	0.0148 (5)	0.0200 (6)	0.0013 (4)	−0.0085 (5)	−0.0058 (5)
O2	0.0176 (5)	0.0137 (5)	0.0161 (6)	0.0002 (4)	−0.0044 (5)	−0.0035 (4)
O3	0.0116 (5)	0.0132 (5)	0.0211 (6)	0.0002 (4)	0.0044 (5)	−0.0038 (4)
O4	0.0150 (5)	0.0170 (5)	0.0174 (6)	0.0032 (4)	0.0002 (5)	−0.0008 (5)
C1	0.0142 (6)	0.0114 (6)	0.0133 (7)	−0.0005 (5)	−0.0010 (6)	−0.0014 (5)
C2	0.0127 (6)	0.0113 (6)	0.0124 (7)	0.0001 (4)	0.0006 (5)	−0.0010 (5)
C3	0.0153 (6)	0.0160 (7)	0.0123 (7)	−0.0008 (5)	−0.0012 (6)	0.0003 (6)
C4	0.0159 (6)	0.0167 (7)	0.0156 (7)	0.0019 (5)	−0.0010 (6)	0.0044 (6)
C5	0.0182 (6)	0.0127 (6)	0.0181 (8)	0.0030 (5)	0.0002 (6)	0.0011 (6)
C6	0.0156 (6)	0.0111 (6)	0.0165 (7)	0.0012 (5)	−0.0008 (6)	−0.0022 (6)
C7	0.0119 (6)	0.0105 (6)	0.0122 (7)	0.0004 (4)	0.0004 (5)	0.0000 (5)
C8	0.0095 (5)	0.0114 (6)	0.0135 (7)	−0.0011 (5)	0.0002 (5)	−0.0017 (5)
C9	0.0097 (5)	0.0099 (6)	0.0125 (6)	0.0008 (4)	0.0002 (5)	−0.0016 (5)
C10	0.0117 (6)	0.0106 (6)	0.0152 (7)	0.0008 (4)	0.0009 (5)	0.0006 (5)
C11	0.0125 (6)	0.0142 (6)	0.0097 (6)	−0.0006 (5)	−0.0007 (5)	−0.0019 (5)
C12	0.0177 (7)	0.0147 (7)	0.0195 (8)	−0.0027 (5)	0.0039 (6)	−0.0004 (6)

### Geometric parameters (Å, °)

**Table:**

O1—C1	1.212 (2)	C5—H5A	0.93
O2—C8	1.217 (2)	C6—C7	1.396 (2)
O3—C9	1.4273 (17)	C6—H6A	0.93
O3—H1O3	0.86 (3)	C7—C8	1.480 (2)
O4—C11	1.2205 (19)	C8—C9	1.540 (2)
C1—C2	1.486 (2)	C9—C10	1.521 (2)
C1—C9	1.542 (2)	C10—C11	1.514 (2)
C2—C3	1.389 (2)	C10—H10A	0.97
C2—C7	1.400 (2)	C10—H10B	0.97
C3—C4	1.397 (2)	C11—C12	1.498 (2)
C3—H3A	0.93	C12—H12A	0.96
C4—C5	1.402 (2)	C12—H12B	0.96
C4—H4A	0.93	C12—H12C	0.96
C5—C6	1.393 (2)		
			
C9—O3—H1O3	104.5 (16)	O2—C8—C9	124.39 (14)
O1—C1—C2	127.44 (16)	C7—C8—C9	108.24 (14)
O1—C1—C9	124.53 (14)	O3—C9—C10	111.50 (12)
C2—C1—C9	108.02 (12)	O3—C9—C8	105.33 (11)
C3—C2—C7	121.55 (13)	C10—C9—C8	114.42 (14)
C3—C2—C1	128.51 (15)	O3—C9—C1	108.50 (13)
C7—C2—C1	109.92 (14)	C10—C9—C1	113.41 (12)
C2—C3—C4	117.60 (15)	C8—C9—C1	103.00 (12)
C2—C3—H3A	121.2	C11—C10—C9	112.60 (12)
C4—C3—H3A	121.2	C11—C10—H10A	109.1
C3—C4—C5	121.03 (15)	C9—C10—H10A	109.1
C3—C4—H4A	119.5	C11—C10—H10B	109.1
C5—C4—H4A	119.5	C9—C10—H10B	109.1
C6—C5—C4	121.14 (14)	H10A—C10—H10B	107.8
C6—C5—H5A	119.4	O4—C11—C12	122.79 (14)
C4—C5—H5A	119.4	O4—C11—C10	120.01 (14)
C5—C6—C7	117.80 (15)	C12—C11—C10	117.16 (13)
C5—C6—H6A	121.1	C11—C12—H12A	109.5
C7—C6—H6A	121.1	C11—C12—H12B	109.5
C6—C7—C2	120.84 (15)	H12A—C12—H12B	109.5
C6—C7—C8	129.16 (15)	C11—C12—H12C	109.5
C2—C7—C8	109.98 (13)	H12A—C12—H12C	109.5
O2—C8—C7	127.35 (14)	H12B—C12—H12C	109.5
			
O1—C1—C2—C3	−1.6 (3)	C2—C7—C8—C9	6.84 (16)
C9—C1—C2—C3	177.10 (15)	O2—C8—C9—O3	−74.26 (18)
O1—C1—C2—C7	176.83 (17)	C7—C8—C9—O3	104.64 (14)
C9—C1—C2—C7	−4.49 (17)	O2—C8—C9—C10	48.55 (19)
C7—C2—C3—C4	0.5 (2)	C7—C8—C9—C10	−132.56 (13)
C1—C2—C3—C4	178.78 (15)	O2—C8—C9—C1	172.10 (14)
C2—C3—C4—C5	1.4 (2)	C7—C8—C9—C1	−9.00 (15)
C3—C4—C5—C6	−2.1 (3)	O1—C1—C9—O3	75.56 (19)
C4—C5—C6—C7	0.8 (2)	C2—C1—C9—O3	−103.16 (14)
C5—C6—C7—C2	1.1 (2)	O1—C1—C9—C10	−48.9 (2)
C5—C6—C7—C8	−177.55 (15)	C2—C1—C9—C10	132.37 (13)
C3—C2—C7—C6	−1.8 (2)	O1—C1—C9—C8	−173.13 (16)
C1—C2—C7—C6	179.63 (14)	C2—C1—C9—C8	8.15 (16)
C3—C2—C7—C8	177.09 (14)	O3—C9—C10—C11	−177.41 (13)
C1—C2—C7—C8	−1.45 (17)	C8—C9—C10—C11	63.19 (17)
C6—C7—C8—O2	4.5 (3)	C1—C9—C10—C11	−54.58 (17)
C2—C7—C8—O2	−174.31 (15)	C9—C10—C11—O4	−16.2 (2)
C6—C7—C8—C9	−174.36 (15)	C9—C10—C11—C12	161.75 (15)

### Hydrogen-bond geometry (Å, °)

**Table:**

*D*—H···*A*	*D*—H	H···*A*	*D*···*A*	*D*—H···*A*
O3—H1O3···O2^i^	0.86 (3)	1.93 (3)	2.7907 (16)	174 (3)
C3—H3A···O4^ii^	0.93	2.51	3.401 (2)	159
C12—H12A···O4^iii^	0.96	2.54	3.408 (2)	150

Symmetry codes: (i) −*x*+1/2, *y*−1/2, *z*−1/2; (ii) *x*, *y*, *z*−1; (iii) −*x*, −*y*, *z*+1/2.
